# Can Annual Daylight Cycles and Seasons Have an Effect on Male Sexual Functions?

**DOI:** 10.7759/cureus.18879

**Published:** 2021-10-18

**Authors:** Mehmet Caniklioğlu, Ünal Öztekin, Ayşen Caniklioğlu, Volkan Selmi, Sercan Sarı, Levent Işıkay

**Affiliations:** 1 Urology, Bozok University Faculty of Medicine, Yozgat, TUR; 2 Urology, Kayseri System Hospital, Kayseri, TUR; 3 Clinical Biochemistry, Bozok University Faculty of Medicine, Yozgat, TUR

**Keywords:** daylight, periodicity, seasons, libido, male sexuality

## Abstract

Introduction

Mammals’ sexual functions exhibit seasonal variations that have been attributed to changes in the daylight. In this study, taking into consideration endocrine and psychogenic status, we aimed to investigate whether human males experience changes in erectile functions and sexual desire depending on daylight periods and seasons, and whether periodicity exists in human sexual behavior.

Materials and methods

International Index of Erectile Function (IIEF) and psychiatric scale scores of 221 male patients were evaluated. In addition, hormonal parameters of the patients were examined. These data were first evaluated in two groups (summer and winter) according to local daylight amounts the participants received. Then IIEF scores were also analyzed according to four conventional seasons (winter, spring, summer, and autumn).

Results

There was no significant difference in laboratory data, psychiatric scale scores and IIEF evaluations between summer and winter groups. Moreover, no significant difference was found in terms of sexual desire and erectile functions in terms of four seasons (p > 0.05).

Conclusion

According to the results of this study, there is no periodicity in human sexual functions both in relation to daylight and four seasons.

## Introduction

Sexual behavior and functions originate from complex neuropsychoendocrine factors. Environmental factors have also been suggested to affect sexual functions [1]. The studies that investigated the effects of seasons and air temperature on sexual behaviors have suggested that sexual behavior differences between summer and winter may be due to the changes in living comfort caused by the change in air temperature or the effects of cloudy versus clear weather on human psychology. In short, our mood, which is affected by environmental or seasonal changes, has an important effect on our sexual desire and behavior [2,3].

In most animals, sexual behavior and reproductive cycles are periodic. Although in mammals, this periodic behavior has been claimed to be related to complex environmental factors, it is mostly associated with certain chemokines and hormones [4]. Humans can reproduce throughout the year and are not considered to be periodic in their sexual activities. However, there are very few studies that investigated this subject. In humans, although hormone levels do not show a significant difference between the seasons, levels of total testosterone (TT), follicular stimulating hormone (FSH), luteinizing hormone (LH) and prolactin were reported to be lower in the winter [2]. Another study reported that TT levels were higher in autumn months compared to other months [5]. In the same study, it is also said that sexual activity can increase in the summer months due to more sunlight exposure.

In this study, taking into consideration endocrine and psychogenic status, we aimed to investigate whether human males experience changes in erectile functions and sexual desire depending on daylight periods and seasons, and whether periodicity exists in human sexual behavior. In this context, this is the first prospective study that investigates the relationship between daylight and male sexual functions.

## Materials and methods

A total of 221 patients who visited our clinic between January 2018 and January 2020 were included in the study. An ethics committee approval was obtained for this case-control study from Yozgat Bozok University, Clinical Trials Ethics Committee (2017-KAEK-189_2020.01.22_1). After obtaining informed consent from all participants, the International Index of Erectile Function (IIEF) and psychiatric scale scores were filled out. Exclusion criteria of the study were age of >45 years, not getting married or lack of regular sexual partner, presence of pathologies that may affect sexual function (hormonal disorders, TT <35 ng / dL, prolactinoma, hypothyroidism, hyperthyroidism), disease that may affect overall performance and psychology (renal failure, coronary and cardiac problems, hepatic insufficiency, non-depression severe psychiatric problems) or missing data related with IIEF scores and hormonal parameters. A total of 49 patients were excluded from the study. Demographic data, hormonal parameters such as prolactin, FSH, LH, estradiol (E2), TT levels, IIEF, psychiatric evaluation scales such as Health Anxiety Inventory (HAI), Beck Depression Inventory (BDI), Beck Anxiety Inventory (BAI), somatosensory amplification scores (SSAS) of the remaining 172 patients were evaluated. The psychiatric scale scores were evaluated for 115 patients that had psychiatric scale scores.

The patients completed the IIEF questionnaire evaluating their sexual activities in the last month and were grouped based on their responses. For the first analysis patients were divided into two groups based on the official daylight quantity they had received. The number of sunny days obtained locally and their distribution by months were consistent with the known solstice dates. Therefore, the time between 24 September and 21 March was accepted as winter months, while summer months were designated as the time between 22 March and 23 September. The average daylight hours for these months were 9.11 hours/day for the summer and 4.43 hours/day for the winter. For the second analysis patients were divided into four groups based on the known seasons as spring, summer, autumn, and winter. Since the number of cases in the autumn group was very low, the second analysis was limited only to erectile function and sexual desire parameters. Psychiatric scale scores were not examined for four seasons.

Patients were requested to fill out all evaluation forms in a peaceful environment when they felt calm. When evaluating the erectile function with the IIEF scale the answers to questions 1-5 and 15 were added and patients with a score between 0-10 were interpreted to have severe, 11-16 moderate, 17-21 mild-moderate, and 22-25 mild erectile dysfunction. Those with a score of 26-30 were considered as having normal erectile function [6].

Sexual desire level was evaluated by summing the scores of IIEF's 11th and 12th questions. Since there is no international scale for the assessment of sexual desire, the evaluations were made only by comparing the numerical data with each other.

The SSAS scale was used to assess patient’ exaggeration of physical senses [7]. In this scale, there are 10 questions with 1-5 points assigned to each answer. The numerical data obtained for each patient were compared.

The clinical anxiety states of the patients were evaluated with the BAI scale, which contains 21 questions with 0-3 points assigned to each answer [8]. The comparison between the groups was done by comparing the obtained numerical data.

The clinical status assessment related to depression was evaluated with BDI [9]. Similar to BAI, the BDI scale consists of 21 questions and scores between 0-9 points are interpreted as having minimal depressive symptoms, 10-18 mild depression, 19-29 moderate, and 30-63 points as severe depression.

The presence of health anxiety in patients was evaluated with HAI [10]. Each of 14 questions of this inventory was evaluated on a scale of 0-3 and the resulting numerical scores were compared with each other.

IBM SPSS Statistics for Windows, v25.0 (IBM Corp. Released 2017. IBM SPSS Statistics for Windows, Version 25.0. Armonk, NY: IBM Corp.) was used for statistical analyses. The distribution properties of the patients’ numerical data were evaluated with the Kolmogorov-Smirnov test. While binary comparisons for parametric data in independent groups were done with t-test, Mann-Whitney U test was applied for non-parametric data. Chi-square test and Fischer exact test were used for binary comparisons of categorical data. The comparisons of four seasons showed parametric distribution, but since the number of cases in the autumn group was only 9, the evaluation was made with the Kruskal-Wallis test. p-value <0.05 was considered significant.

## Results

Demographic and laboratory test data of the patients are summarized in Table 1. There was no significant difference between summer and winter groups in terms of either demographic or laboratory data.

**Table 1:** Demographic and laboratory data of the patients in terms of daylight exposure. The data were given in mean±SD for numerical data and (n, %) for categorical data in the table. For a better presentation and due to larger amounts of standard deviation values of marriage time parameter, it is given as median (minimum – maximum). Some missing values for categorical parameters were not listed in the table. There was no statistically significant difference between the groups. BMI: body mass index; FSH: follicular-stimulating hormone; LH: luteinizing hormone; E2: estradiol; T.T.: total testosterone. *p < 0.05.

Parameter		Summer (n=87)	Winter (n=85)	p-value
Age (year)		30.11 ± 4.85	29.94 ± 5.38	0.75
Marriage time (year)		2 (0.3 – 25)	2 (0.4 – 32)	0.65
Age of spouse (year)		25.73 ± 4.22	26.61 ± 6.30	0.77
BMI (kg/m^2^)		26.26 ± 3.87	26.86 ± 4.17	0.33
Smoking	Present	40 (48.8%)	50 (59.5%)	0.16
	Absent	42 (51.2%)	34 (40.5%)	
Morning erections	Present	81 (98.8%)	84 (100%)	0.49
	Absent	1 (1.2%)	0 (0%)	
Impaired libido (due to medical history)		3 (3.6%)	4 (4.8%)	1.00
Prolactin (µg/L)		10.25 ± 6.00	11.71 ± 18.92	0.81
FSH (mIU/ml)		4.97 ± 3.96	4.29 ± 3.50	0.21
LH (IU/L)		4.02 ± 2.05	3.56 ± 1.53	0.38
E2 (mIU/ml)		24.11 ± 10.32	26.57 ± 11.31	0.14
TT (ng/dL)		446.36 ± 184.88	454.47 ± 169.64	0.58

The mean age of the patients in the summer and winter groups were 30.11 ± 4.85 and 29.94 ± 5.38 years, respectively. Their spouses' ages were 25.73 ± 4.22 and 26.61 ± 6.30 years, respectively. The marriage duration was 2 (0.3 - 25) years in the summer group and 2 (0.4 - 32) years in the winter group. In the light of this information, it is seen that the patient population consisted of sexually active young adults in the first years of marriage.

While body mass index (BMI) was 26.26 ± 3.87 kg/m^2^ in the summer group, this value was 26.86 ± 4.17 in the winter group (p = 0.33). Smoking rates were high in our study group with 48.8% (n = 40) in the summer group and 59.5% (n = 50) in the winter group. The data of 8 patients could not be accessed. However, there was no significant difference between the two groups in terms of smoking rates (p = 0.16).

The patients’ preliminary examination did not show any significant sexual problems. In the summer group, morning erections were reported at 98.8% and low libido at only 3.6%. In the winter group, these rates were 100% and 4.8%, respectively. Morning erection and libido data of 6 patients could not be reached.

Prolactin, FSH, LH and TT levels were within the normal reference ranges in both groups and there was no significant difference between the two groups (p > 0.05). Although statistically insignificant, TT levels were inversely proportional to FSH and LH levels and were higher in the winter group compared to the summer group. While this level was 446.36 ± 184.88 ng/dL in the summer group, it was 454.47 ± 169.64 ng/dL in the winter group.

The mean scores of scales used in the patients' evaluation are summarized in Table 2. Accordingly, there was no significant difference between the summer and winter seasons in terms of patients' erectile functions (p > 0.05). The mean IIEF score in the summer group was 26.09 ± 4.31, while in the winter group it was 26.61 ± 4.27. Moreover, similar to the libido, there were no significant issues with patients’ sexual desire levels and no significant difference was detected between the two groups (p > 0.05).

**Table 2:** IIEF and psychiatric scale scores data of the patients in terms of daylight exposure. The data were given in mean±SD for numerical data and (n, %) for categorical data. There was no statistically significant difference between the groups. There were only 115 patients that had available psychiatric scale scores. Therefore, the table was divided into two parts. IIEF: International Index of Erectile Function; SSAS: somatosensory amplification scale; HAI: health anxiety inventory; BAI: beck anxiety inventory; BDI: beck depression inventory. *p < 0.05.

Parameters	Summer (n=87)	Winter (n=85)	p-value
IIEF (1-5, 15)	26.09 ± 4.31	26.61 ± 4.27	0.55
IIEF (11-12)	7.73 ± 1.40	7.53 ± 1.57	0.61
	(n=39)	(n=76)	
SSAS	25.76 ± 6.41	24.93 ± 8.15	0.57
HAI	18.46 ± 5.83	17.86 ± 6.93	0.47
BAI	12.92 ± 5.36	14.06 ± 8.66	0.86
BDI	13.12 ± 7.80	12.69 ± 6.89	0.76

Evaluation of the data of 115 patients with available psychiatric scale scores showed that there was no significant difference between the groups (p > .05). While the mean SSAS score was 25.76 ± 6.41 in the summer group, it was 24.93 ± 8.15 in the winter group. In the same order, the scores for HAI were 18.46 ± 5.83 and 17.86 ± 6.93, for BAI they were 12.92 ± 5.36 and 14.06 ± 8.66, and for BDI they were 13.12 ± 7.80 and 12.69 ± 6.89. The multi-eyed Chi-square test for BDI indicated that there was no significant difference between the two groups (p > 0.05).

The evaluations regarding the patients’ erectile functions and sexual desires in terms of four seasons are summarized in Table 3. Since the number of patients filling out the IIEF form in the autumn was quite low (n = 9) compared to other groups, non-parametric analysis was performed and there was no significant difference between the four seasons in terms of both erectile functions and sexual desire (p > 0.05).

**Table 3:** Comparison of some IIEF parameters in terms of four seasons. The data were expressed as mean±SD for numerical data. There was no statistically significant difference between the groups. IIEF: International Index of Erectile Function. *p < 0.05.

Seasons	IIEF (1-5, 15)	IIEF (11-12)
Spring (n=51)	26.20 ± 4.4	7.44 ± 1.2
Summer (n=51)	26.51 ± 3.7	7.91 ± 1.5
Autumn (n=9)	26.67 ± 4.6	8.25 ± 1.4
Winter (n=60)	26.25 ± 4.7	7.47 ± 1.6
p-value	0.99	0.21

## Discussion

The sexual life of mammals follows a periodic behavior. Humans can breed and engage in sexual intercourse throughout the year and thus are thought to be excluded from this patterned behavior. However, studies evaluating seasonality or period specificity in humans’ sexual behavior are limited.

Pheromones play a significant role in the periodicity of sexual behavior in mammals. These molecules are known to be an important communication tool not only in the sexual behavior, but also in mother-offspring relationships and regional domination reporting [4]. Pheromones were first described as external substances that enable communication between insects [11]. Later, mammals were also shown to secrete similar substances and perceive them by smell and taste organs; however, in mammals these molecules were associated with more flexible behavioral models [4].

It has been suggested that mammals that perceive pheromones as odor molecules are affected by daily light-dark periods and their sensitivity to the pheromones of the opposite sex change accordingly. For example, it has been shown that female rats prefer female scents throughout the winter to male scents [12]. Another study reported that exposure to light increases fertilization efficiency [5]. These factors would explain the seasonal changes in animal sexual behavior.

Sexual desire and partner selection are more complicated in humans. First of all, it is debated whether pheromones and body odor, which are thought to play a role in such a mechanisms, have effects on human sexual behavior. In their review, Lanfranchi et al. concluded that pheromones are effective in humans, just as in animals [13]. Sorokowska et al. determined that expression of major histocompatibility complex (MHC) genes had an effect on spouse selection and sexual behavior, and that oral contraceptive use disrupted this communication [14]. Stern et al. reported that they could change the menstrual cycles by applying the samples of female bodily fluids to the upper lip and interpreted that pheromones are also effective in humans [15]. Sergeant MJT stated that adult males can announce their healthy immune system functions and body symmetries to females via their body odors [16]. He also stated that human leukocyte antigen complex (HLA) may be playing a role in this ability. According to him, females were able to perceive these odors and when choosing a spouse preferred males that had opposite HLA characteristics and were more symmetrical [16].

There were also studies that suggested that pheromones have no effect on humans. Havlicek et al. wrote a review on this subject and argued that further studies are needed because the data related to smell selection and facial symmetry are still not sufficient [17]. In a study evaluating married couples, Pollack et al. reported no relationship between the choice of spouse and parameters such as body scent and HLA expression [18].

In this study, we investigated whether there are differences in sexual desire and erection in healthy adult men based on the length of daylight exposure through pheromones or other mechanisms. We divided our evaluation periods based on autumn and spring solstices, which have equal day and night hours. According to our results, we did not find periodicity in human sexual functions. Preliminary evaluation did not reveal decrease in libido, and the IIEF indicated normal erectile function and sexual desire. We also saw that there was no difference in the erectile function and sexual desire values ​​in patients examined in terms of four seasons (p <0.05). Psychogenic factors, hormones, stress factors, visual and auditory stimuli rather than pheromones direct human sexual impulses [1]. Although there are contrary opinions, visual and auditory stimuli stimulate the amygdala more than the pheromones [4,13]. This can be explained by the fact that the vomeronasal organ is rudimentary in humans and the accessory olfactory system is much smaller than the visual and auditory system [4].

Hormonal factors, especially TT levels, have important effects on male sexual functions. Loss of erection, lack of sexual desire, and loss of morning erections are considered as the first symptoms of TT deficiency [19]. In mammals, an increase in cortisol and TT levels has been shown during mating periods [20]. Although there are studies of annual changes by age in humans, studies related to the annual cycle of the hormone profile are limited. Demir et al. divided patients living in a cold climate into two groups according to their temperature levels [2]. According to their results, testosterone levels were within normal limits in both seasons and testosterone levels were lower in cold months than the hot months. Therefore, they claimed that testosterone levels could change according to the season. They suggest considering cold seasons in the evaluation of testosterone levels, sexual status and social or cultural factors of the patients. On the other hand, Kontula et al. suggested that increasing daylight in the summer can increase sexual activity [3]. Moskovic et al. did not find a seasonal change in free and total testosterone levels [21]. In our study, no significant difference was found in any of the examined hormonal parameters both in terms of daylight exposure and in terms of 4 seasons. Moskovic et al. reported seeing seasonal differences in TT/E2 in the population under the age of 60, and when we adapted their data into our database we did not find any difference both in terms of daylight and in terms of 4 seasons (p values ​​0.29 and 0.68, respectively) [21]. Although more studies are needed in this regard, the adrenal glands that have a significant effect on sexual cycle and lactation in animals do not have such a substantial role in humans and it may explain the lack of periodic behavior in humans [20,22].

Several studies have shown that the increase in BMI may result in disruption of hormonal and seminal parameters [23,24]. Therefore, we considered BMI as a parameter that may affect the results in our study; however, we found that there was no difference in BMI between our groups. Our study’s participants were all in the normal BMI range. Therefore, we think that the results we obtained are quite suitable for making a comparison in the hormonal and psychological factors.

In a large study by Nimbi et al., male sexual behavior was shown to be influenced by a very wide etiological spectrum that includes psychosocial factors [1]. Brotto et al. also drew attention to the psychosocial aspects of sexual dysfunction and emphasized the need for multidisciplinary approach to deal with this issue [25]. It does not seem rational to evaluate the sexual life and cycles of humans, who have such advanced cortical functions and socio-psychological interactions, by excluding brain capacity. Therefore, in our study, we took into account the psychological states of our human subjects. Psychiatric scale scoring has been used for many years with many variations [26]. We have extensive clinical experience with scales such as SSAS, HAI, BAI and BDI in our clinic. Based on our psychiatric scoring data, excluding BDI, we can say that our patients had low levels of health anxiety, overall anxiety as well as exaggeration of their physical sensations and therefore comprised a study population without any pathological problems. However, the results of BDI scale indicated that 82.4% of our patients have depressive complaints ranging from mild to severe. The number of patients describing moderate or severe depression was 19 (10.8%). Under normal circumstances, we would expect worse IIEF scores in such a population, but we found that our patients had a fairly healthy erectile function score of 26.31 ± 4.38 in the whole population. Sexual desire score of the whole population was also good with 7.63 ± 1.48. Regardless of the results, we found that there was no difference between the daylight groups in terms of all psychiatric scale scores and IIEF scores. Although our patients showed significant depressive symptoms in the psychiatric sense, these symptoms were not significantly different between the seasons, and patients’ sexual cycles were also not affected by various seasons.

The novelty of this study is assessing the periodicity of sexual life of human males quantitatively using several tools like indexes, laboratory findings and scales including psychological ones. Also, this is the first study that evaluates sexual periodicity of human beings in terms of daylight exposure like in other animal studies. When we look at the literature, this is the first comprehensive prospective study that investigates the effect of daylight on male sexual functions, however, there are several limitations. Correlation analysis between the psychiatric scale scores obtained by filling out provided questionnaires with the results of psychiatric evaluation by trained professionals would have provided us with a more accurate assessment. Lack of this correlation analysis is one limitation of our study. Another limitation of this study is the lack of data such as occupation, exercise regimen, social activities and partner satisfaction status that can directly affect the amount of light patients can receive during the day. Moreover, the educational status of our patients could not be analyzed due to lack of data. Whether educational status has an effect on the periodicity of sexual behavior would be an interesting subject to investigate. As is known, in general, the free forms of the hormones are responsible for their physiological effects [27]. Whether free and total testosterone rates show periodicity could also have been evaluated in this study. However, due to the lack of ability to measure free testosterone in our hospital we were not able to evaluate these parameters. Also, a study that includes semen analyses, and which compares pregnancy rates beside those parameters in our study would give information about the reproduction status of this population.

## Conclusions

According to the results of this study, we have concluded that there is no periodicity in human sexual life, either seasonally or based on daylight exposure. We think that even if pheromones have effects on human sexual life, they do not cause any periodicity. In this study, participants did not show any significant difference in terms of BMI, hormonal or psychiatric evaluation. Erectile functions and libido did not change throughout the year. Our study population consisted of healthy, sexually active, young individuals that did not have any factors that could negatively impact sexual function or desire, therefore we consider this study as valuable addition to the literature. Since our study was conducted only in the male population, it is obvious that there is a need for prospective randomized studies that include sexual partners.
